# The effect of flavanol-rich cocoa on cerebral perfusion in healthy older adults during conscious resting state: a placebo controlled, crossover, acute trial

**DOI:** 10.1007/s00213-015-3972-4

**Published:** 2015-06-07

**Authors:** Daniel J. Lamport, Deepa Pal, Christina Moutsiana, David T. Field, Claire M. Williams, Jeremy P. E. Spencer, Laurie T. Butler

**Affiliations:** School of Psychology and Clinical Language Sciences, University of Reading, Reading, RG6 6AP UK; Molecular Nutrition Group, School of Chemistry, Food and Pharmacy, University of Reading, Reading, RG6 6AP UK

**Keywords:** Flavonoid, Flavanol, Cocoa, Arterial spin labelling, Cerebral blood flow, Cognition, Regional perfusion, Imaging, fMRI

## Abstract

**Rationale:**

There has recently been increasing interest in the potential of flavanols, plant-derived compounds found in foods such as fruit and vegetables, to ameliorate age-related cognitive decline. Research suggests that cocoa flavanols improve memory and learning, possibly as a result of their anti-inflammatory and neuroprotective effects. These effects may be mediated by increased cerebral blood flow (CBF), thus, stimulating neuronal function.

**Objectives:**

The present study employed arterial spin labelling functional magnetic resonance imaging to explore the effect of a single acute dose of cocoa flavanols on regional CBF.

**Methods:**

CBF was measured pre- and post-consumption of low (23 mg) or high (494 mg) 330 ml equicaloric flavanol drinks matched for caffeine, theobromine, taste and appearance according to a randomized counterbalanced crossover double-blind design in eight males and ten females, aged 50–65 years. Changes in perfusion from pre- to post-consumption were calculated as a function of each drink.

**Results:**

Significant increases in regional perfusion across the brain were observed following consumption of the high flavanol drink relative to the low flavanol drink, particularly in the anterior cingulate cortex and the central opercular cortex of the parietal lobe.

**Conclusions:**

Consumption of cocoa flavanol improves regional cerebral perfusion in older adults. This provides evidence for a possible acute mechanism by which cocoa flavanols are associated with benefits for cognitive performance.

## Introduction

In recent years, there has been a great deal of interest in the neuroprotective effects of flavonoids, with evidence emerging that they may lead to improvements in memory and learning by improving neuronal functioning and promoting neuronal protection and regeneration (Spencer 2009). One sub-class of flavonoids which has attracted interest in this respect are flavanols. Dietary intervention trials have shown that consumption of flavanol-containing cocoa products are associated with a range of health benefits such as reduced platelet aggregation (Holt et al. 2002), improved insulin sensitivity (Grassi et al. 2005) and reduced blood pressure (Taubert et al. 2007).

Further to the aforementioned memory and learning effects, randomised placebo-controlled trials indicate that daily consumption of flavanol-rich cocoa drinks over 8 weeks is associated with improved cognitive function in healthy older adults (Mastroiacovo et al. 2015) and older adults with mild cognitive impairment (Desideri et al. 2012). Human trials have predominately revealed that tests of executive function and processing are sensitive to cocoa flavanols (see Scholey and Owen 2013, for a review). More specifically, cocoa drinks containing 990 and 520 mg flavanols were associated with benefits for executive function and processing speed relative to a 48-mg flavanol cocoa drink. Furthermore, there is evidence that a single dose of cocoa flavanols can also confer positive cognitive effects in the immediate post-prandial period, even in healthy young populations. For example, Field et al. (2011) observed better spatial working memory and choice reaction time 2 h following consumption of dark chocolate containing 773 mg flavanols relative to a white chocolate control, whilst Scholey et al. (2010) observed improvements in executive function 1 h after consumption of 990 or 520 mg cocoa flavanols relative to a 46-mg control. Interestingly, positive effects for mood were also reported, such that self- reported increases in mental fatigue over the morning were attenuated by the 520 mg flavanol drink. Similar benefits for subjective mood in the form of increased calmness and contentedness have also been observed following 30 days of daily consumption of 550 mg cocoa flavanols, although no behavioural effects were reported (Pase et al. 2013).

The evidence suggests that consumption of flavanol-rich cocoa is associated with observable improvements in behavioural outcomes. It has been postulated that vascular effects form the basis of the mechanisms by which flavanol ingestion may lead to benefits for neuronal and cognitive function (Spencer 2009). For example, there is evidence of sub-chronic and acute increases in peripheral blood flow following ingestion of cocoa (Faridi et al. 2008), which may translate to increased cerebral blood flow (CBF). Francis et al. (2006) observed an increase in CBF (increased global perfusion in grey matter) using arterial spin labelling (ASL) in a pilot study of four healthy young females following consumption of a 516-mg cocoa flavanol drink relative to a 39-mg flavanol cocoa drink, which peaked approximately 2 h after consumption. Interestingly, in a separate sample of 16 healthy young females, an increase in the blood oxygenation level-dependent (BOLD) signal (measured with functional magnetic resonance imaging (fMRI)) was observed in various brain regions during performance of an attention switching task following daily consumption of 172 mg cocoa flavanols relative to 13 mg over 5 days using a parallel groups design (Francis et al. 2006). However, there was no evidence that the changes in the BOLD signal were associated with cognitive benefits since the performance on the attention switching task was not different between the high and low flavanol conditions. Similarly, Camfield et al. (2012) reported regional amplitude modulations in steady-state visually evoked potentials measured with Steady State Probe Topography during memory encoding in a spatial working memory task following 30 days, daily consumption of 250 and 500 mg cocoa flavanol drinks relative to a low flavanol placebo. Whilst no behavioural effects on the spatial working memory task were observed, the authors suggested that the observed amplitude modulations indicate more efficient task performance.

More recently, promising data has emerged showing a coupling between cerebral physiology and cognitive benefits following cocoa flavanol consumption. Brickman et al. (2014) observed increased cerebral blood volume (CBV) in a sub-region of the hippocampus (dentate gyrus) as measured with fMRI following ingestion of a daily 900 mg flavanol supplement (*n* = 21) over 3 months relative to a 45-mg flavanol supplement (*n* = 20) in healthy older adults (aged 50–69). Crucially, significantly faster pattern recognition was observed during the fMRI assessment for the high flavanol intervention relative to the low flavanol intervention (with baseline performance included as a covariate). Moreover, the positive correlation between CBV and faster reaction time indicates that increases in CBF in the hippocampus might also be associated with behavioural benefits on hippocampal-dependent memory tasks.

Whilst there is some evidence to support benefits for CBF following chronic cocoa flavanol consumption, only one study has investigated acute effects within the immediate postprandial period (Francis et al. 2006) which is a small pilot study (*n* = 4) that only reports changes in global CBF. Brickman et al. (2014) provide evidence for regional perfusion benefits following chronic (3-month) ingestion of cocoa flavanols; therefore, it is of interest to examine whether regional effects are also observed following acute cocoa flavanol administration, particularly, given that behavioural benefits have been documented within this timeframe (e.g. Scholey et al. 2010; Field et al. 2011). Following from this, the aim of the present study was to investigate perfusion on a regional basis in the human brain with ASL in the acute postprandial period following cocoa flavanol consumption.

## Materials and methods

### Participants

Eighteen (10 males) native English-speaking healthy older adults aged 55–65 years (mean age 61 years) without neurological symptoms were recruited from the local community. Exclusion criteria included obesity (>30 kg/m^2^), current or recent illness, diagnosed diabetes, diagnosed cardiovascular disease, diagnosed hypertension, history of stroke, chronic consumption of medication, gall bladder/gastrointestinal abnormalities, high habitual consumption of alcohol (15 units/week), consumption of illegal substances, evidence of dementia (Mini-Mental State Examination; MMSE <26), language or hearing impediments and allergy or sensitivity to chocolate, dairy, nuts or gluten. Inclusion/exclusion criteria were checked during a screening session with a standard medical questionnaire. The study was conducted according to the guidelines of the Declaration of Helsinki, and all procedures involving human subjects were approved by the University of Reading Research Ethics committee. All participants provided written informed consent at the screening visit.

### Design

A 2 × 2 randomised counterbalanced crossover double-blind design was employed with two conditions, low flavanol (LF) and high flavanol (HF), and two time points, baseline and 2 h. An independent researcher generated a random allocation sequence (using restricted randomisation) and allocated participants into their groups. The order of drink consumption was counterbalanced such that nine participants consumed the LF at visit one and nine consumed the HF at visit one. The alternate drink was consumed at visit two following a 1-week washout.

### Drinks

The 330-ml intervention drinks were provided by Mars Incorporated (Hackettstown, NJ, USA) as dry, dairy-based cocoa beverage mixes. The low and high flavanol drinks contained 23 and 494 mg total flavanols, respectively (see Table 1). The amount of total cocoa flavanols referenced here is defined as the sum of all monomeric flavanols and their oligomeric derivatives (dimers to decamers, i.e. 2–10 monomeric subunits). Both drinks were equicaloric (113 kcal) and matched for macronutrients, micronutrients, theobromine (HF, 185 mg; LF, 177 mg) and caffeine (HF, 15 mg; LF, 17 mg). The LF and HF drinks were matched for taste and appearance by the manufacturers and were supplied in individual sachets labelled with an anonymous three-digit code; thus, all experimenters and participants remained blinded during the procedures and analysis. The code was broken following completion of the analysis.

**Table 1:** Composition of high flavanol and low flavanol cocoa drinks

Component	High cocoa flavanol drink (HF)	Low cocoa flavanol drink (LF)
Packet size, g	30	30
Cocoa flavanols (DP 1–10), mg	494	29
Epicatechin, mg	89	3
Catechin, mg	21	3
Dimers–decamers, mg	384	20
Calories	113	112
Total fat, g	1	1
Saturated fat, g	1	1
Cholesterol, mg	5	5
Sodium, mg	197	204
Total carbohydrates, g	16	16
Dietary fiber, g	3	4
Sugars, g	10	9
Protein, g	9	9
Caffeine, mg	15	17
Theobromine, mg	185	176
Potassium, mg	507	573

### Procedure

Initially, telephone screening interviews were performed and volunteers who met the inclusion criteria were invited to attend the Centre for Integrative Neuroscience and Neurodynamics (CINN) for a screening visit. At the screening visit, inclusion/exclusion criteria were checked with the MMSE and a standard medical questionnaire, and informed consent was obtained following a description of the study procedures. Each test visit was identical. Participants arrived at 8 a.m. having fasted from 8 p.m. the previous night (water consumption was permitted). Prior to each test day visit, participants were also instructed to avoid vigorous physical activity and polyphenol-rich foods for 24 h (including berries, fruits, fruit juices, jams and preserves, red wine, black, green and fruit teas, coffee, cocoa, soy products, caffeinated energy drinks and vegetables, except potatoes) and were provided with standardised typed instructions identifying which foods to avoid. Upon arrival, a standardized breakfast of croissants and low fat cream cheese was consumed (24.5 g fat, 10.1 g protein, 38.4 g carbohydrates and 415 kcal). Following breakfast, participants completed an initial 12-min scan including structural and ASL sequences detailed below. For each scan, participants were instructed to close their eyes without sleeping and to remain as still as possible. Upon completion of the scan, participants consumed the 330-ml-cocoa beverage. Two hours post-ingestion, a second 12-min scan was completed. During the 2 h between scans, participants remained within the CINN where they were permitted to read and only consume water.

### fMRI Protocol

Scanning was performed at the CINN, University of Reading, UK using a 3.0 T Siemens MAGNETOM Trio MRI scanner with a 12-channel Head Matrix coil. The pulsed ASL images were acquired using the PICOREQ2T sequence with the following parameters: number of slices = 18, slice thickness = 5.0 mm, inter-slice gap = 1.25 mm, TR = 2500 ms, TE = 11 ms, TI1 = 700, Saturation Stop Time = 1,600, TI2 = 1,800 and perfusion mode = PICOREQ2T (pulsed). A high-resolution whole-brain three-dimensional anatomical image was also acquired using an MPRAGE-gradient sequence with 176 × 1 mm thick slices (1 × 1 × 1 voxels size; TE, 2.52 ms; TR, 2,020 ms; TI, 1,100 ms; FOV, 250 × 250; slice thickness, 2 mm; and flip angle, 9°). FMRI data processing was carried out using FEAT (FMRI Expert Analysis Tool) Version 5.98, part of FSL (FMRIB’s Software Library, www.fmrib.ox.ac.uk/fsl). ASL volumes from each scanning session were all registered to the corresponding individual’s high resolution structural image using rigid body transformations. In a second step, the images were registered to the Montreal Neurological Institute (MNI) template brain using a 12° of freedom affine transformation algorithm. To allow voxelwise comparisons we processed each CBF map individually by using perfusion signal modelling, which models the differences between control images and tagged (spin labelled) images within a time series.

### Statistical analysis

It was hypothesised that there would be an increase in perfusion between the two time points (baseline and 2 h) only for the HF condition. This hypothesis was tested by contrasting the two difference maps (i.e. perfusion as change from baseline for the HF drink contrasted with perfusion as change from baseline for the LF drink). In order to do, this a CBF map was produced for each participant, time point (baseline and 2 h) and drink (LF and HF). These perfusion flow maps were then provided as inputs to the second level analyses which, separately for each drink, processed the difference between the 2-h post-drink and the pre-drink baseline. Specifically, these contrasts had the form of a simple subtraction defined as: CBF 2 h − CBF baseline. The output of this second step was voxelwise contrast images which corresponded to the change in the perfusion flow post-drink consumption. The second level contrast images were then entered in a third level analysis consisting of a paired sample *t* test comparing the drink conditions. Specifically, change in perfusion after the HF drink was contrasted with change in perfusion after the LF drink. The resulting *Z* (Gaussianised T/F) statistic image was then cluster thresholded with initial clusters determined using a voxelwise uncorrected height threshold of *Z* > 2.3 followed by a cluster significance threshold of *p* < 0.05 (corrected for multiple comparisons). Prior to analysis, normality checks were performed on all data. Analyses were performed with FSL v4.0.

## Results

**Fig. 1:**
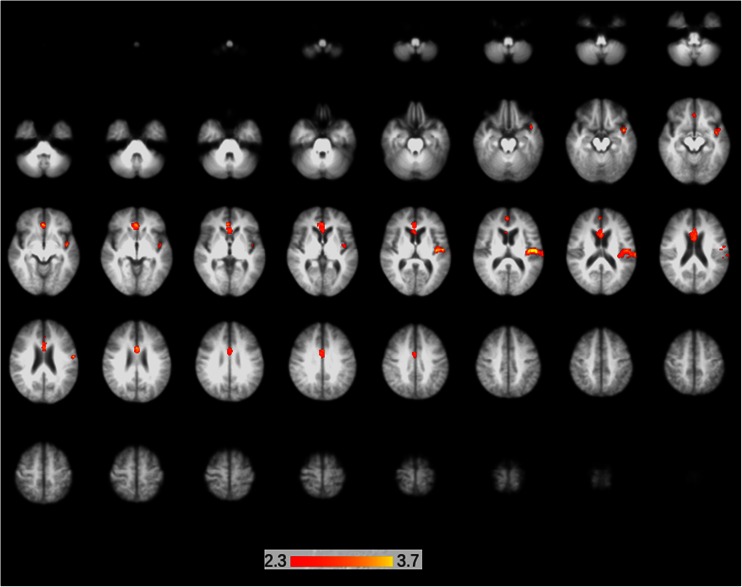
Axial slices showing regions of significantly greater regional perfusion 2 h post-consumption of the high flavanol drink relative to the low flavanol drink using arterial spin labelling. Significantly greater change in blood perfusion occurred following the high flavanol drink compared to the low flavanol drink in two clusters; the ACC and the central opercular cortex of the left parietal lobe down to a sub-cluster in the temporal pole. Anatomical labels and coordinates are given in Table 2. Activations are superimposed on axial slices of the MNI template brain. The images were initially thresholded at *Z* > 2.3 to identify activation clusters and then a (corrected) cluster significance threshold of *p* < 0.05 was applied (see the “Materials and methods” section)

The HF–LF contrast analysis revealed significantly increased regional perfusion 2 h post-consumption of the HF drink relative to the LF drink. As shown in Fig. 1, there are two main clusters of voxels with significantly greater CBF at 2 h post-consumption of the HF drink. The first cluster is in the anterior cingulate cortex (ACC), with a sub-cluster slightly posterior and superior to the main focus. The second cluster extends from the central opercular cortex of the left parietal lobe down to a sub-cluster in the temporal pole. The details of this, including coordinates and cluster sizes, are given in Table 2. Importantly, the reverse contrast, low flavanol–high flavanol, produced no activation. The reverse contrast was performed because significant activation would have been inconsistent with the hypothesis.

**Table 2:** Stereotaxic coordinates (MNI template brain) and cluster sizes for brain areas where significantly greater change in blood perfusion occurred following the high flavanol drink (494 mg) relative to the low flavanol drink (23 mg)

Brain region	No. of voxels	*X*	*Y*	*Z*	Peak *Z* stat
Anterior cingulate	1,040	0	34	−4	3.9
Anterior cingulate (peak of sub-cluster)		0	12	28	3.5
Left parietal lobe: central opercular cortex	864	−44	−14	−4	3.8
Peak of sub-cluster: temporal pole		−42	6	18	2.7

## Discussion

The present data provide evidence that consumption of cocoa flavanols is associated with acute increases in regional CBF in older adults as measured with ASL. More specifically, statistically significant increased perfusion was observed in the anterior cingulate cortex and regions of the parietal lobe 2 h post-consumption of 494 mg cocoa flavanols in the form of a drink relative to a calorie, caffeine and theobromine-matched cocoa drink containing 23 mg flavanols. These findings are consistent with the only previously published study of acute effects of a single cocoa flavanol dose on blood flow in the brain using ASL. Francis et al. (2006) demonstrated increased global CBF 2 h post-consumption of 516 mg cocoa flavanols in small sample of young adults. The present data extends these findings by demonstrating increased perfusion in specific brain regions in older adults.

The present findings are also consistent with functional imaging data showing regional specific increases in the BOLD response to a task following daily consumption of 172 mg cocoa flavanols relative to 13 mg over 5 days (Francis et al. 2006). More specifically, Francis et al. (2006) observed increased BOLD response in the dorsolateral prefrontal cortex, parietal cortex and ACC, whilst presently, increased perfusion was observed in the ACC and the central opercular cortex of the left parietal lobe down to a sub-cluster in the temporal pole. In the study by Francis et al. (2006), participants performed an executive function task during the BOLD assessment; therefore, the regions of activity assessed were specifically associated with task switching. In contrast, the present findings show increased perfusion during conscious resting state. Despite the different natures of the two studies (BOLD during task vs. ASL at rest), it is notable that there is some regional overlap in the ACC between these two studies, highlighting the particular sensitivity of the ACC to flavanol ingestion. As part of the brain’s limbic system, the ACC plays a role in environmental and task monitoring, distributing attention and learning and is activated in a wide variety of cognitive and emotional neuroimaging tasks (for the review, see Bush et al. 2000).

As no task was performed in the current study, it is difficult to make associations between the presently observed regions of increased perfusion and previously demonstrated behavioural effects. Following a single dose of cocoa, cognitive improvements in the immediate postprandial period have been shown for executive function (Scholey et al. 2010), spatial memory and attention (Field et al. 2011), although interpretations directly related to flavanols from the latter study are limited due to a non-matched white chocolate control. To advance this field, direct associations between increased CBF/activation and behavioural outcomes are required. Indeed, Brickman et al. (2014) provide evidence for a direct association between increased CBV in the dentate gyrus and improved performance on a hippocampal-dependent pattern recognition task following 3 months daily cocoa flavanol consumption. However, similar evidence following a single cocoa dose is required. Other studies have failed to find simultaneous increases in CBF or neuronal activation and improved cognitive performance. For example, Francis et al. (2006) reported an increase in CBF during an attention switching task following 172 mg cocoa flavanols relative to 13 mg, but attention switching performance was not affected. Similarly, increased steady state evoked potentials (assessed using Steady State Probe Topography) in posterior parietal and central–frontal regions was observed in middle-aged adults following 30 days daily consumption of 250 or 500 mg cocoa flavanol drinks relative to placebo; however, there were no effects for behavioural measures of spatial working memory (Camfield et al. 2012). Clearly, further studies are required concomitantly examining CBF and neuronal activation with behavioural cognitive measures following both long term and acute consumption of cocoa flavanols.

The specific mechanisms underlying regional specific perfusion remain to be elucidated; however, there is now a growing body of evidence supporting a beneficial effect of cocoa flavanols for peripheral (Schroeter et al. 2006) and cerebral vascular function in humans. Flavanol-rich cocoa has been shown to induce vasodilation via activation of the nitric oxide system (Fisher et al. 2003), providing one plausible mechanism for vascular benefits. Moreover, cocoa flavanols have been associated with enhancement of several measures of endothelial function and nitric oxide synthesis in animal (Karim et al. 2000) and human models (Fisher et al. 2003; Heiss et al. 2003; Fisher and Hollenberg 2006). Recently, a meta-analysis of the effects of cocoa on endothelial function reported a pooled estimate increase of 2 % in a flow-mediated dilation following cocoa consumption relative to placebo/control (Petrone et al. 2013).

Interestingly, in one study, cocoa flavanol induced improvement in endothelial function was greatest in older adults relative to younger adults (Fisher and Hollenberg 2006), which raises the possibility that effects of cocoa flavanols on CBF may vary as a function of age. Age mediated effects could explain why Francis et al. (2006) observed increased global perfusion in younger adults, whereas the present study in older adults revealed increased regional perfusion; however, it should be acknowledged that the former study did not analyse or report regional perfusion and therefore direct comparisons with the present data are limited. Nevertheless, future research should investigate the effect of age since it may shed light on underlying mechanisms. If increased vasodilation via nitric oxide synthesis accounts for the vascular benefits (Fisher et al. 2003), then older adults with poorer endothelial function may be more likely than younger adults with efficient endothelial function to benefit from cocoa flavanol consumption. For example, populations including smokers (Heiss et al. 2005) and overweight adults (Faridi et al. 2008) have shown improved endothelial vasodilation following cocoa flavanol ingestion.

In order for cocoa flavanols and their metabolites to exert effects on the brain, they would be expected to cross the blood brain barrier (BBB; see Nehlig et al. 2012 for review). Rodent models have demonstrated that epicatechin is found in the brain following oral ingestion (Abd El Mohsen et al. 2002) leading to increased dendritic spine density in the dentate gyrus (van Praag et al. 2007). Various flavonoid-binding sites on neurons have now been described: adenosine (Agullo et al. 1997), GABAA (Johnston 2005; Fernandez et al. 2008), δ-opioid (Katavic et al. 2007; Panneerselvam et al. 2010), nicotinic (Lee et al. 2010; Lee et al. 2011), TrkB (Jang et al. 2010), estrogen (Jacobson et al. 2002) and testosterone receptors (Bastianetto et al. 2010) and, more generally, a specific brain plasma membrane binding site for polyphenols has been proposed (Goyarzu et al. 2004). At present, it is unclear which, if any, of these receptors is responsible for mediating flavanol-induced stimulation of neuronal activity which could account for increased blood flow in specific regions (Miller et al. 2001; Wang et al. 2003; Tjandra et al. 2005), and furthermore, it is difficult to establish cause and effect between increased neuronal activity and increased regional specific blood flow. However, receptor-binding flavanols and their metabolites will trigger the activation of various downstream kinases, including various members of both the MAP kinase and PI3 kinase pathways, and as well as inducing the opening of agonist-activated ion channels leading to increased activity (Luh et al. 2000; Kim and Duong 2002). Further work is required in order to identify which receptors are modulated by flavanols and to what extent this occurs.

There is now good evidence that the acute benefits for cognitive function and blood flow exerted by cocoa flavanol consumption peak approximately 90–120 min post-consumption (Schroeter et al. 2006; Francis et al. 2006; Scholey et al. 2010; Field et al. 2011); however, it is presently unclear whether separate chronic mechanisms exists following cumulative consumption over several weeks and months, or indeed whether chronic consumption enhances the effectiveness of acute mechanisms in a cumulative fashion. Despite several plausible mechanisms for increased neuronal activity (as described above), it remains to be seen whether a single cocoa flavanol dose-induced increase in CBF is associated with concomitant benefits in cognitive performance in the immediate postprandial period. More broadly, recent reviews of acute interventions and epidemiological surveys provide good evidence that flavonoids and their subclasses are beneficial for cognitive function (Macready et al. 2009; Lamport et al. 2012; Scholey and Owen 2013). Investigations of CBF and behavioural effects should also be extended to other flavanol-rich foods and beverages (e.g. tea, grapes and apples). For example, consumption of apples containing 180 mg epicatechin and 184 mg quercetin glycosides has been shown to acutely augment nitric oxide status (Bondonno et al. 2014), a potential mechanism by which flavanols may lead to increased CBF; however, there were no effects on behavioural outcomes. Increased neuronal activation has also been observed acutely using electroencephalogram measurements following a 300-mg epigallocatechin gallate dose (commonly found in tea; Scholey et al. 2012). In relation to this, future studies should consider the effects of habitual flavonoid intake since it is possible that benefits following an acute or chronic intervention will only be observed when habitual intake is low, although this was not considered presently.

In conclusion, the present findings support the hypothesis that flavanol-rich cocoa beverages are associated with increased CBF within a 2-h post-prandial time frame. More specifically, increased brain perfusion following the HF drink relative to the LF drink was observed in the anterior cingulate cortex and a region in the left parietal lobe. These data add to the substantial body of literature demonstrating that flavanol consumption is beneficial for peripheral and cerebral vascular function and thus for maintaining, protecting and enhancing cardiovascular health.

## References

[CR1] Abd El Mohsen MM, Kuhnle G, Rechner AR, Schroeter H, Rose S, Jenner P, Rice-Evans CA (2002). Uptake and metabolism of epicatechin and its access to the brain after oral ingestion. Free Radic Biol Med.

[CR2] Agullo G, Gamet-Payrastre L, Manenti S, Viala C, Rémésy C, Chap H, Payrastre B (1997). Relationship between flavonoid structure and inhibition of phosphatidylinositol 3-kinase: a comparison with tyrosine kinase and protein kinase C inhibition. Biochem Pharmacol.

[CR3] Bastianetto S, Dumont Y, Duranton A, Vercauteren F, Breton L, Quirion R (2010). Protective action of resveratrol in human skin: possible involvement of specific receptor binding sites. PLoS One.

[CR4] Bondonno CP, Downey LA, Croft KD, Scholey A, Stough C, Yang X, Considine MJ, Ward NC, Puddey IB, Swinny E, Mubarak A, Hodgson JM (2014). The acute effect of flavonoid-rich apples and nitrate rich spinach on cognitive performance and mood in healthy men and women. Food Funct.

[CR5] Brickman AM, Khan UA, Provenzano FA, Yeung L-K, Suzuki W, Schroeter H, Wall M, Sloan RP, Small SA (2014). Enhancing dentate gyrus function with dietary flavanols improves cognitive in older adults. Nat Neurosci.

[CR6] Bush G, Luu P, Posner MI (2000). Cognitive and emotional influences in anterior cingulate cortex. Trends Cog Sci.

[CR7] Camfield DA, Scholey A, Pipingas A, Silberstein R, Kras M, Nolidin K, Wesnes K, Pase M, Stough C (2012). Steady state visually evoked potential (SSVEP) topography changes associated with cocoa flavanol consumption. Physiol Behav.

[CR8] Desideri G, Kwik-Uribe C, Grassi D, Necozione S, Ghiadoni L, Mastroiacovo D, Raffaele A, Ferri L, Bocale R, Carmela L, Marini C, Reffi C (2012). Benefits in cognitive function, blood pressure, and insulin resistance through cocoa flavanol consumption in elderly subjects with mild cognitive impairment: the cocoa cognition and ageing (CoCoA) study. Hyperten.

[CR9] Faridi Z, Njike VY, Dutta S, Ali A, Katz DL (2008). Acute dark chocolate and cocoa ingestion and endothelial function: a randomized controlled crossover trial. Am J Clin Nutr.

[CR10] Fernandez SP, Mewett KN, Hanrahan JR, Chebib M, Johnston GA (2008). Flavan-3-ol derivatives are positive modulators of GABA(A) receptors with higher efficacy for the alpha(2) subtype and anxiolytic action in mice. Neuropharmacol.

[CR11] Field DT, Williams CM, Butler LT (2011). Consumption of cocoa flavanols results in an acute improvement in visual and cognitive functions. Physiol Behav.

[CR12] Fisher ND, Hollenberg NK (2006). Aging and vascular responses to flavanol-rich cocoa. J Hypertens.

[CR13] Fisher ND, Hughes M, Gerhard-Herman M, Hollenberg NK (2003). Flavanol-rich cocoa induces nitric-oxide-dependent vasodilation in healthy humans. J Hypertens.

[CR14] Francis ST, Head K, Morris PG, Macdonald IA (2006). The effect of flavanol-rich cocoa on the fMRI response to a cognitive task in healthy young people. J Cardiovasc Pharmacol.

[CR15] Goyarzu P, Malin DH, Lau FC, Taglialatela G, Moon WD, Jennings R, Moy E, Moy D, Lippold S, Shukitt-Hale B, Joseph JA (2004). Blueberry supplemented diet: effects on object recognition memory and nuclear factor-kappa B levels in aged rats. Nutr Neurosci.

[CR16] Grassi D, Necozione S, Lippi C, Croce G, Valeri L, Pasqualetti P, Desideri G, Blumberg JB, Ferri C (2005). Cocoa reduces blood pressure and insulin resistance and improves endothelium-dependent vasodilation in hypertensives. Hypertension.

[CR17] Heiss C, Dejam A, Kleinbongard P, Schewe T, Sies H, Kelm M (2003). Vascular effects of cocoa rich in flavan-3-ols. JAMA.

[CR18] Heiss C, Kleinbongard P, Dejam A, Perre S, Schroeter H, Sies H, Kelm M (2005). Acute consumption of flavanol-rich cocoa and the reversal of endothelial dysfunction in smokers. J Am Coll Cardiol.

[CR19] Holt RR, Schramm DD, Keen CL, Lazarus SA, Schmitz HH (2002). Chocolate consumption and platelet function. Am Med Assoc.

[CR20] Jacobson KA, Moro S, Manthey JA, West PL, Ji XD (2002). Interactions of flavones and other phytochemicals with adenosine receptors. Adv Exp Med Biol.

[CR21] Jang SW, Liu X, Yepes M, Shepherd KR, Miller GW, Liu Y, Wilson WD, Xiao G, Blanchi B, Sun YE, Keqiang YE (2010). A selective TrkB agonist with potent neurotrophic activities by 7, 8-dihydroxyflavone. Proc Natl Acad Sci U S A.

[CR22] Johnston GA (2005). GABA (A) receptor channel pharmacology. Curr Pharma Des.

[CR23] Karim M, McCormick K, Kappagoda CT (2000). Effects of cocoa extracts on endothelium-dependent relaxation. J Nutr.

[CR24] Katavic PL, Lamb K, Navarro H, Prisinzano TE (2007). Flavonoids as opioid receptor ligands: identification and preliminary structure-activity relationships. J Nat Prod.

[CR25] Kim SG, Duong TQ (2002). Mapping cortical columnar structures using fMRI. Physiol Behav.

[CR26] Lamport DJ, Dye L, Wightman JD, Lawton CL (2012). The effects of flavonoid and other polyphenol consumption on cognitive performance: a systematic research review of human experimental and epidemiological studies. Nutr Aging.

[CR27] Lee BH, Choi SH, Shin TJ, Pyo MK, Hwang SH, Kim BR, Lee SM, Lee JH, Kim HC, Park HY (2010). Quercetin enhances human alpha7 nicotinic acetylcholine receptor-mediated ion current through interactions with Ca(2+) binding sites. Mol Cells.

[CR28] Lee BH, Choi SH, Shin TJ, Pyo MK, Hwang SH, Lee SM, Paik HD, Kim HC, Nah SY (2011). Effects of quercetin on alpha9alpha10 nicotinic acetylcholine receptor-mediated ion currents. Eur J of Pharmacol.

[CR29] Luh WM, Wong EC, Bandettini PA, Ward BD, Hyde JS (2000). Comparison of simultaneously measured perfusion and BOLD signal increases during brain activation with T (1)-based tissue identification. Magn Reson Med.

[CR30] Macready AL, Kennedy OB, Ellis JA, Williams CM, Spencer JPE, Butler LT (2009). Flavonoids and cognitive function: a review of human randomized controlled trial studies and recommendations for future studies. Genes Nutr.

[CR31] Mastroiacovo D, Kwik-Uribe C, Grassi D, Necozione S, Raffaele A, Pistacchio L, Righetti R, Bocale R, Lechiara MC, Marini C, Ferri C, Desideri G (2015). Cocoa flavanol consumption improves cognitive function, blood pressure control and metabolic profile in elderly subjects: the cocoa cognition, and ageing (CoCoA) study – a randomised controlled trial. Am J Clin Nutr.

[CR32] Miller KL, Luh WM, Liu TT, Martinez A, Obata T, Wong EC, Frank LR, Buxton RB (2001). Nonlinear temporal dynamics of the cerebral blood flow response. Hum Brain Mapp.

[CR33] Nehlig A (2012). The neuroprotective effects of cocoa flavanol and its influence on cognitive performance. Br J Clin Pharm.

[CR34] Panneerselvam M, Tsutsumi YM, Bonds JA, Horikawa YT, Saldana M, Dalton ND, Head BP, Patel PM, Roth DM, Patel HH (2010). Dark chocolate receptors: epicatechin-induced cardiac protection is dependent on delta-opioid receptor stimulation. Am J Physiol Heart Circ Physiol.

[CR35] Pase MP, Scholey AB, Pipingas A, Kras M, Nolidin K, Gibbs A, Wesnes K, Stough CK (2013). Cocoa polyphenols enhance positive mood states but not cognitive performance. A randomized, placebo-controlled trial. J Psychopharm.

[CR36] Petrone AB, Gaziano JM, Djousse L (2013). The effects of dark chocolate and cocoa products on endothelial function: a meta analysis. Curr Nutr Rep.

[CR37] Scholey A, Downey LA, Ciorciari J, Pipingas A, Nolidin K, Finn M, Wines M, Catchlove S, Terrens A, Barlow E, Gordon L, Stough C (2012). Acute neurocognitive effects of epigallocatechin gallate (EGCG). Appetite.

[CR38] Scholey A, Owen L (2013). Effects of chocolate on cognitive function and mood: a systematic review. Nutr Rev.

[CR39] Scholey A, French SJ, Morris PJ, Kennedy DO, Milne AL, Haskell CF (2010). Consumption of cocoa flavanols results in acute improvements in mood and cognitive performance during sustained mental effort. J Psychopharmacol.

[CR40] Schroeter H, Heiss C, Balzer J, Kleinbongard P, Keen CL, Hollenberg NK, Sies H, Kwik-Uribe C, Schmitz HH, Kelm M (2006). (−)-Epicatechin mediates beneficial effects of flavanol-rich cocoa on vascular function in humans. Proc Natl Acad Sci.

[CR41] Spencer JPE (2009). Flavonoids and brain health: multiple effects underpinned by common mechanisms. Genes Nutr.

[CR42] Taubert D, Roesen R, Lehmann C, Jung N, Schömig E (2007). Effects of low habitual cocoa intake on blood pressure and bioactive nitric oxide. A randomized controlled trial. JAMA.

[CR43] Tjandra T, Brooks JC, Figueiredo P, Wise R, Matthews PM, Tracey I (2005). Quantitative assessment of the reproducibility of functional activation measured with BOLD and MR perfusion imaging: implications for clinical trial design. Neuroimage.

[CR44] van Praag H, Lucero MJ, Yeo GW, Stecker K, Heivand N, Zhao C, Yip E, Afanador M, Schroeter H, Hammerstone J, Gage FH (2007). Plant-derived flavanol (−)epicatechin enhances angiogenesis and retention of spatial memory in mice. J Neurosci.

[CR45] Wang J, Aguirre GK, Kimberg DY, Roc AC, Li L, Detre JA (2003). Arterial spin labeling perfusion fMRI with very low task frequency. Magn Reson Med.

